# Current Clinical Trials for the Monkeypox Virus

**DOI:** 10.1007/s44229-023-00029-y

**Published:** 2023-03-07

**Authors:** Mahmoud Kandeel

**Affiliations:** 1 Department of Biomedical Sciences, College of Veterinary Medicine, King Faisal University, Al-Hofuf, 31982 Al-Ahsa Saudi Arabia; 2 Department of Pharmacology, Faculty of Veterinary Medicine, Kafrelshikh University, Kafrelshikh, 33516 Egypt

**Keywords:** Monkeypox, Virus, Clinical trials, Vaccine, Treatment

## Abstract

**Background:**

Monkeypox (MPX) is a zoonotic Orthopoxvirus causing smallpox-like symptoms. Before April 2022, MPX cases outside Africa were rare. The virus can spread through skin-to-skin contact, sexual contact, respiratory droplets, and household items such as towels and blankets.

**Aim:**

This study was aimed at highlighting the dire need for vaccination and treatment against this infection. Several in-process clinical trials that may help overcome MPX infection are discussed.

**Methods:**

A search for recent clinical studies was conducted in the clinicaltrials.gov database.

**Results:**

A total of 15 trials were identified. After February 2022, 14 new trials were launched. Of the 15 trials, 9 were observational studies, 3 were treatment studies and 3 were preventive studies. MPX clinical trial topics were classified into four broad categories: MPX virus shedding and clearance; response to MPX vaccine; antiviral treatment for MPX; and awareness regarding MPX. One medication, tecovirimat, and two vaccines are currently in clinical trials.

**Conclusions:**

Few treatments and vaccines are under evaluation. Although multiple trials have been conducted, evidence to determine the present state of MPX infection is currently insufficient. Global collaboration is required to achieve complete understanding of the epidemiology, prevention and control of MPX.

## Introduction

Although the fight against SARS-CoV-2 is still ongoing worldwide, public health officials are worried about the reemergence of an monkeypox outbreak [1]. Orthopoxvirus is the most important genus of the family Poxviridae, which contains a variety of viruses dangerous to humans [2]. For instance, the smallpox-causing variola virus killed between 300 and 500 million people in the twentieth century [3].

The MPX virus was first discovered in monkeys, but it also naturally infects tree squirrels, rope squirrels, Gambian pouched rats and dormice [4]. Several incidences of this novel endemic have been considered to be associated with sexual contact, particularly in bisexual or males [5, 6]. Additionally, MPX can spread via body fluids, infected sores, scabs, and sharing of bedding and clothing [7]. Symptoms of infection caused by MPX include a typical rash followed by less prodromal signs. However, these signs are less severe than those of smallpox (e.g., high body temperature, lymphadenopathy and flu-like symptoms) [8]. The current MPX manifestations are atypical, beginning with a normal rash in the vaginal and perianal areas, and expanding to other body parts. After the prodrome or rash appears, individuals are regarded as contagious until the lesions scab, and the scabs fall off. The ideal method for determining whether a case of active MPX is present is testing for viral DNA in swabs collected from blister or ulcer crusts [9]. MPX cases began to occur worldwide at the beginning of May 2022, thus indicating a shift in the disease's epidemiological range toward previously nonendemic areas [10]. Most of these instances were in men having sex with men, without a prior history of travel to endemic areas. Most recent infections, in contrast to earlier outbreaks, have been documented among homosexual, bisexual and other men who have sex with men [11].

According to the Centers for Disease Control and Prevention, no particular medications are available for patients with MPX viral infection, and supportive care is often sufficient [12, 13]. Smallpox vaccines and antiviral agents have been effective in controlling smaller outbreaks [14]. MPX prevention and treatment measures are similar to those for other orthopoxviral infections. The ACAM2000 and JYNNEOS vaccines have been licensed by the FDA to prevent Orthopoxvirus infection. JYNNEOS is approved for only MPX, and ACAM2000 is approved for only smallpox but may be effective in preventing MPX. Vaccines may potentially be provided after exposure to prevent disease, owing to the long incubation time of MPX and the rapid development of antibodies after immunization [15–17].

Three antiviral medications—cidofovir, brincidofovir and tecovirimat—can combat MPX and other orthopoxviruses [18]. The DNA replication inhibitor cidofovir and its prodrug brincidofovir are effective against many different types of double-stranded DNA viruses [19]. To prevent the production of the extracellular enveloped virus required for cell-to-cell transmission, tecovirimat has been shown to have a more targeted effect on orthopoxviruses [18, 20, 21].

## Trial Search and Summary of Prior Research

The clinical trials website clinicaltrials.gov was searched for currently registered clinical trials. A summary of the search findings is provided in Table 1. A total of 15 trials were identified, 14 of which started after February 2022. The retrieved 15 trials comprised 9 observational, 3 treatment and 3 prevention randomized clinical trials. The trial topics comprised four main categories: MPX virus shedding and clearance, response to MPX vaccine, antiviral treatment for MPX and awareness regarding MPX.

**Table 1:** Current clinical trials on the monkeypox virus. The data are from the clinicaltrials.gov website

#	NCT #	Title	Intervention	Age	Phase	Enrollment	Study type	Study design	Start date	Completion date
1	NCT05522296	Break-through Infection Following Monkeypox Vaccination	Biological: monkeypox vaccine	18 years or older		4638	Observational	Observational model: cohort|time perspective	13/09/2022	13/09/2023
2	NCT05559099	Tecovirimat for Treatment of Monkeypox Virus	Drug: tecovirimat oral capsule|drug: placebo	Child, adult	Phase 2	450	Interventional	Randomized|intervention model: parallel assignment|masking: purpose: treatment	10/10/2022	01/09/2024
3	NCT05476744	Viral Clearance and Epidemiological Characteristics in Patients with Monkeypox		18 years or older		75	Observational	Observational model: cohort|time perspective: prospective	28/06/2022	01/04/2023
4	NCT05562323	Characterization of Vaccine-induced Responses Against Monkeypox (MoVIHvax) An Observational Prospective Cohort Study		18 years or older		100	Observational	Observational model: cohort|time perspective: prospective	14/09/2022	23/04/2023
5	NCT05443867	Monkeypox Asymptomatic Shedding: Evaluation by Self-Sampling MPX-ASSESS		18 years or older		140	Observational	Observational model: cohort|time perspective: prospective	22/06/2022	31/12/2022
6	NCT05534984	Study of Tecovirimat for Human Monkeypox Virus	Drug: tecovirimat oral capsule|drug: placebo|drug: tecovirimat oral capsule (open label)	Child, adult	Phase 3	530	Interventional	Randomized|intervention model: parallel assignment|masking: purpose: treatment	12/09/2022	30/09/2023
7	NCT05534165	Tecovirimat in Non-hospitalized Patients with Monkeypox	Drug: tecovirimat|drug: placebo	18 years or older	Phase 3	120	Interventional	Randomized|intervention model: parallel assignment|masking: purpose: treatment	01/09/2022	01/02/2023
8	NCT05543577	Assessing the Preparedness and Knowledge of Pharmacists in the Current Monkeypox Outbreak	Other: questionnaire	Child, adult		380	Observational	Observational model: other|time perspective: cross-sectional	14/09/2022	01/12/2022
9	NCT05567939	Clinical, Virological, Immunological, Psychosocial and Epidemiological Consequences of Human Monkeypox Virus (ProMPX)	Other: natural course of disease	16 years or older		300	Observational	Observational model: cohort|time perspective: prospective	19/09/2022	19/12/2023
10	NCT05438953	Follow-up of Contact at Risk of Monkeypox Infection: a Prospective Cohort Study	Biological: vaccination with MVA vaccine ( IMVANEX® and JYNNEOS®)	18 years or older	Not applicable	226	Interventional	Non-randomized|intervention model: parallel assignment|masking: none|purpose: prevention	12/07/2022	01/07/2023
11	NCT02977715	IMVAMUNE® Smallpox Vaccine in Adult Healthcare Personnel at Risk for Monkeypox in the Democratic Republic of the Congo	Biological: IMVAMUNE	18 years or older	Phase 3	1600	Interventional	N/A|intervention model: single group assignment|masking: none|purpose: prevention	23/02/2017	01/08/2022
12	NCT05058898	A One Health Study of Monkeypox Human Infection	Other: blood samples|other: scabs and pus samples	Child, adult		280	Observational	Observational model: case–control|time perspective: prospective	09/09/2021	01/12/2023
13	NCT05512949	Trial to Evaluate the Immunogenicity of Dose Reduction Strategies of the MVA-BN Monkeypox Vaccine	Biological: JYNNEOS	18 years to 50 years	Phase 2	210	Interventional	Randomized|intervention model: parallel assignment|masking: none|purpose: prevention	07/09/2022	13/12/2023
14	NCT05597735	A Phase III Trial to Assess the Efficacy and Safety of Tecovirimat in Patients with Monkeypox Virus Disease	Drug: tecovirimat|drug: placebo	14 years or older	Phase 3	150	Interventional	Randomized|intervention model: parallel assignment|masking: purpose: treatment	01/12/2022	01/01/2025
15	NCT03745131	Cohort Study of Healthcare Workers Receiving Imvanex®	Other: blood draw	18 years or older		120	Observational	Observational model: cohort|time perspective: prospective	30/10/2018	30/11/2019

## Current Ongoing Clinical Trials on MPX Virus

### MPX Virus Shedding and Clearance

#### Viral Clearance and Epidemiological Characteristics in Patients with MPX (NCT05476744)

By measuring MPX viral load over time in various specimens, a 75-participant observational prospective cohort study is being undertaken to better understand the dynamics of viral clearance among patients with confirmed MPX. A selection of participants will also undergo an immunological study to describe humoral and cellular reactions to MPX. The study remains in progress and is expected to yield fruitful outcomes.

#### MPX Asymptomatic Shedding: Evaluation by Self-sampling (NCT05443867)

This clinical study involving 140 individuals is being conducted in Belgium. This research aims to perform a monitored follow-up investigation of close contacts of confirmed MPX cases. The participants are individuals with high-risk relationships with people with confirmed cases of MPX who sought a diagnosis. At the time of enrollment, contacts exhibiting symptoms consistent with MPX infection according to national case definitions will be recruited. Until 21 days after contact, contacts of the index case who exhibit symptoms at recruitment or develop symptoms during follow-up will be asked to provide specimens at various time points as suspected cases.

#### A One Health Study of MPX Human Infection (NCT05058898)

The goals of this study is to 1) better understand the epidemiology of monkeypox in the Central African Republic by determining the animal reservoir, 2) describing the human outbreak in clinical and epidemiological detail, 3) taking an ethnological approach to the factors that put people at risk, 4) taking a more ecological look at the conditions that led to the disease's emergence, 5) improving biological diagnostics. This multidisciplinary case–control study with 280 participants is aimed at understanding the epidemiology of MPX in the Central African Republic. This research is aimed at investigating the MPX disease outbreak by using a nationwide surveillance infrastructure.

### Response to MPX Vaccine

#### Characterization of Vaccine-Induced Responses Against MPX (NCT05562323)

This 100-participant observational cohort perspective study was financed by BCN Checkpoint and IrsiCaixa, and is being conducted in Spain. The study's goal is to examine the humoral and cellular immune responses in people with human immunodeficiency virus (HIV) after MPX vaccination.

In this trial, individuals with HIV who receive MPX vaccination for pre-exposure prophylaxis fall into two categories: HIV positive and HIV negative individuals. The study seeks to investigate humoral and cellular immune responses in HIV-positive or negative people at high risk of MPX infection during the ongoing MPX vaccination program. The primary outcomes are the proportion of individuals with detectable anti-MPX antibodies and the quantities of the measured antibodies.

#### Break-Through Infection After MPX Vaccination (NCT05522296)

An observational cohort prospective analysis with a goal follow-up time of 6 months in 4638 individuals is being conducted to measure and control the confounders in the presence of a shared cause between the vaccine and the outcome event. The experiment will use a biological MPX vaccine administered in a single intradermal dose of 0.1 ml of live modified vaccinia virus Ankara (MVA).

Participants will include people who visit sexual health clinics in Barcelona or Madrid, or MPX vaccine clinics. People who consent to participate, and meet all the inclusion criteria and no exclusion criteria, will complete a baseline self-reported survey collecting sociodemographic data and details regarding risk factors, sexual activity, vaccination and exposure to MPX.

Participants who have and have not received vaccines will be paired according to criteria associated with infection risk. Each participant's follow-up will end after the following events have occurred: infection with MPX, vaccination (for untreated controls), vaccination of the corresponding control (for treated individuals) or the end of the study, to verify the self-reporting and confirm the diagnosis in new cases.

#### Follow-up of Contacts at Risk of MPX Infection (NCT05438953)

The goal of this research is to predict the percentage of at-risk MPX contacts who do not mount a protective immune response after receiving a single dose of the modified MVA vaccine. Currently, information regarding the efficacy of post-exposure prophylaxis with MVA is lacking. The Centers for Disease Control and Prevention believes that post-exposure vaccination with 2 doses of the MVA vaccine does not completely prevent infection, but that rapid vaccination of high-risk contacts may lessen the severity of symptoms. Establishing a national cohort of contacts of cases meeting the indications for vaccination, i.e., those seen within 14 days after the last contact, has been recommended to better understand the clinical effects and safety of the post-exposure vaccine.

#### IMVAMUNE^®^ Smallpox Vaccine in Adult Healthcare Personnel at Risk of MPX in the Democratic Republic of the Congo (NCT02977715)

A collaboration between the Democratic Republic of the Congo's Ministry of Public Health, the Kinshasa School of Public Health and the Bavarian Nordic has resulted in this clinical trial, an open label prospective cohort study including as many as 1600 qualified healthcare staff regularly exposed to MPX. In addition to documenting participant exposure to, and infection with, MPX, the study will assess the immunogenicity and safety of the vaccine IMVAMUNE^®^ among medical staff. Male and female healthcare workers in Tshuapa Province in the Democratic Republic of the Congo who are at risk of contracting the MPX virus through routine work, or test center employees conducting MPX virus investigative testing are eligible to participate in the study voluntarily. The percentage of participants who (1) develop suspected or confirmed MPX infection and (2) are exposed to the MPX virus after receiving the vaccine will be assessed. IMVAMUNE's immunogenicity and safety will also be examined.

#### Trial to Evaluate the Immunogenicity of Dose Reduction Strategies of the MVA-BN MPX Vaccine (NCT05512949)

This study was sponsored by the National Institute of Allergy and Infectious Diseases. In this phase 2 randomized, open-label, non-placebo controlled, multi-site clinical trial, healthy vaccinia-naive adults 18–50 years of age will be included. The trial will compare two intradermal treatments with the modified vaccinia Ankara-Bavarian Nordic (MVA-BN) vaccine to the conventional subcutaneous regimen.

At least 210 participants will be randomly assigned to the three study arms. One-fifth (2 × 10^7^) and one-tenth (1 × 10^7^) of the usual dosage of MVA-BN will be delivered intradermally on days 1 and 29, respectively, as part of the two dose-sparing method (arms 1 and 2, respectively). The two-dose conventional (1 × 10^8^) MVA-BN s/c regimen will serve as the comparator arm (arm 3).

The purpose of the study is to: (1) examine whether the effects of an i/d schedule on the final humoral immune response of 2 × 10^7^ TCID50 MVA-BN are non-inferior to the licensed regimen of 1 × 10^8^ MVA-BN administered s/c, and (2) determine whether massive humoral immune responses after an i/d regimen of 1 × 10^7^ TCID50 MVA-BN are non-substandard to the registered regimen.

### Antiviral Treatment for MPX

#### Tecovirimat for Treatment of MPX Virus (NCT05559099)

Adults and children with laboratory-confirmed MPX virus illness at study locations in the Democratic Republic of the Congo will be offered tecovirimat oral capsules as part of an interventional, randomized, placebo-controlled, double-blind trial. After the initial 28 days, participants can choose to return for a follow-up session on day 59 for a more in-depth evaluation of their progress. The number of capsules and the dosing schedule will depend on each participant's weight, as follows: (1) 120 kg: three capsules three times per day (total daily tecovirimat dose: 1,800 mg); (2) 40–120 kg: three capsules twice per day (total daily tecovirimat dose: 1,200 mg); (3) 25–40 kg: two capsules twice per day (total daily tecovirimat dose: 800 mg); (4) 13–25 kg: two capsules per day (total daily tecovirimat dose: 400 mg); (5) 6–13 kg: 100 mg twice daily (total daily tecovirimat dose: 200 mg); and (6) 4–6 kg: 50 mg twice daily (total daily tecovirimat dose: 100 mg).

#### Study of Tecovirimat for Human MPX Virus (NCT05534984)

A clinical investigation is being performed by the National Institute of Allergy and Infectious Diseases (NIAID) and SIGA Technologies. The trial is a double-blind, randomized, placebo-controlled study to determine the effectiveness of tecovirimat for treating participants with laboratory-confirmed or suspected MPX illness.

A total of 530 eligible and informed volunteers will be randomized 2:1 to tecovirimat or a placebo. Open-label tecovirimat will be given to participants with severe illness, major skin problems or severe immune suppression. Open-label tecovirimat will be administered to pregnant or nursing participants after a discussion of the advantages and disadvantages. Tecovirimat will be given in an open label manner to participants under the age of 18 years.

After enrollment, participants will receive medication for 14 days. Participants will keep a daily log of their symptoms, self-monitor their skin and mucosal lesions for 29 days or until they disappear (whichever comes first), complete a daily numerical rating scale for pain assessment and keep a daily diary of their symptoms. Participants will attend weekly visits for MPX disease assessment, safety evaluations, MPX sampling similar to that performed at study entry, and swabbing of new MPX lesions. On day 57, patients will be examined for any signs of infection recurrence or the development of new lesions after the first remission of the disease.

#### Tecovirimat in Non-hospitalized Patients with MPX (NCT05534165)

Marina Klein is performing this clinical trial at the McGill University Health Centre in conjunction with the Research Institute of the McGill University Health Centre, the University Health Network, Toronto Unity Health, and the University of British Columbia and the Canadian HIV Trials Network.

The parallel collaborative trial PLATINUM-CAN is associated with the sister trial PLATINUM, led by Oxford University. The PLATINUM CAN feasibility trial lacks the power to assess the primary outcome of time to active lesion resolution. Therefore, results from the sibling trial, PLATINUM-UK (*N* = 500), undertaken at Oxford University, UK, of identical design will be integrated with those for the entire study through a planned individual patient meta-analysis. The trial will assess the relationship between the time to active and complete clearance of lesions according to self-reported and blinded photographic validation by an adjudication committee in consenting participants, and will examine feasibility outcomes.

#### Tecovirimat Treatment for Orthopox Virus Exposure (NCT02080767)

The U.S. Army Medical Research and Development Command funded the ongoing clinical trial. Adult or pediatric patients (40 kg) will be chosen on the basis of the interpretation of the most recent data on human safety and PK data as well as animal efficacy. The therapeutic regimen is the same as the smallpox treatment: three capsules of 200 mg ingested two times per day for 14 days, 30 min after consumption of food containing approximately 600 kcal and 30% (25 g) fat. Participants will be advised to take missed doses as soon as possible unless it is almost time for the following dose. In the event of a missed dose, the prescribed amount should not be doubled. Treatment may be continued for more than 14 days with the sponsor's consent, if the investigator considers it necessary. Use for up to 90 days is supported by nonclinical safety evidence.

Physical vitality, such as body weight, blood pressure, heart rate, respiration rate, temperature and height will be assessed. Medical history and concurrent medications will also be noted. If lesions appear, pictures of the affected areas may be obtained. After the last dose of tecovirimat, patients will be monitored for at least 30 days or until the infection clears up.

### Awareness of MPX

#### Assessing the Preparedness and Knowledge of Pharmacists in the Current MPX Outbreak (NCT05543577)

Beni-Suef University is the sponsor of this clinical investigation. The objective of the study involving 380 participants is to estimate the level of knowledge regarding the nature of the disease and management of MPX, as well as the preparedness for, and perceptions of, the disease among pharmacists and medical interns.

## Completed Clinical Trial

### Cohort Study of Healthcare Workers Receiving Imvanex^®^ (NCT03745131)

Imvanex, a third-generation smallpox vaccine, was first administered to healthcare professionals who came into contact with cases of MPX or were anticipated to do so during the 2018 MPX outbreak in the UK. This study compared the antibody responses in these healthcare workers versus controls to demonstrate that the vaccination induced a response compatible with protection in the context of the trial. Public Health England and Bavarian Nordic collaborated to support the study. The trial applied Imvanex^®^ (MVA-BN, Bavarian Nordic GmBH; also known as Imvamune^®^), a third-generation smallpox vaccine, in cohorts of people for pre-exposure or post-exposure prophylaxis against MPX during an MPX outbreak in the UK. The study was aimed at demonstrating that vaccination-induced antibodies neutralize the specific MPX viruses involved in the U.K. outbreak and reference MPX viruses, and at assessing anti-vaccinia antibody responses to the vaccine. This study measured and characterized antibody responses to Imvanex^®^ delivered in a non-trial context, thus marking the first use of Imvanex^®^ as a public health intervention for an MPX outbreak.

## Conclusion

While the world fights to control the COVID-19 pandemic, reports of an MPX outbreak are increasing. Two vaccines have been developed to protect against this virus to date. Several clinical studies already underway were described herein. The treatment interventions involved tecovirimat clinical trials, and other strategies included decreasing the dose of an existing MPX vaccine. The number of clinical trials remains suboptimal to adequately respond to this dangerous emerging infection. More research on the MPX viral disease is urgently needed to prevent its global spread as a pandemic.

## Abbreviations

MPX: Monkeypox
HIV: Human immunodeficiency virus
MVA: Vaccinia virus Ankara
MVA-BN: Modified vaccinia Ankara-Bavarian Nordic

## Declarations

### Availability of data and materials

All data are within the manuscript.

## References

[1] Yang Z (2022). Monkeypox: a potential global threat?. J Med Virol.

[2] Shchelkunov SN (2013). An increasing danger of zoonotic orthopoxvirus infections. PLoS Pathog.

[3] Babkin IV, Babkina IN, Tikunova NV (2022). An update of Orthopoxvirus molecular evolution. Viruses.

[4] Guarner J, Del Rio C, Malani PN (2022). Monkeypox in 2022—what clinicians need to know. JAMA.

[5] Wieder-Feinsod A, Zilberman T, Erster O, Kolasko GW, Biber A, Gophen R, Hoffman T, Litchevsky V, Olmer L, Yahav D (2023). Overlooked monkeypox cases among men having sex with men during the 2022 outbreak—a retrospective study. Int J Inf Dis.

[6] Ferdous J, Barek MA, Hossen MS, Bhowmik KK, Islam MS (2023). A review on monkeypox virus outbreak: new challenge for world. Health Sci Rep.

[7] Cabanillas B, Murdaca G, Guemari A, Torres MJ, Azkur AK, Aksoy E, Vitte J, de Las VL, Giovannini M, Fernández-Santamaria R (2023). A compilation answering 50 questions on Monkeypox virus and the current Monkeypox outbreak. Allergy.

[8] Pal M, Gutama KPJAJMR (2022). Emergence of monkeypox raises a serious challenge to public health. Cureus..

[9] Durski KN, McCollum AM, Nakazawa Y, Petersen BW, Reynolds MG, Briand S, Djingarey MH, Olson V, Damon IK, Khalakdina AJWER (2018). Emergence of monkeypox in West Africa and Central Africa, 1970–2017. MMWR Morb Mortal Wkly Rep.

[10] Liu X, Zhu Z, He Y, Lim JW, Lane B, Wang H, Peng Q, Sun L, Lu H (2022). Monkeypox claims new victims: the outbreak in men who have sex with men. Infect Dis Poverty.

[11] Peiró-Mestres A, Fuertes I, Camprubí-Ferrer D, Marcos MÁ, Vilella A, Navarro M, Rodriguez-Elena L, Riera J, Català A, Martínez MJ et al. Frequent detection of monkeypox virus DNA in saliva, semen, and other clinical samples from 12 patients, Barcelona, Spain, May to June 2022. Eurosurveillance. 2022; 27. 10.2807/1560-7917.ES.2022.27.28.2200503 PMC9284919 35837964

[12] Khalil A, Samara A, O’Bbrien P, Morris E, Draycott T, Lees C, Ladhani SJTLGH (2022). Monkeypox vaccines in pregnancy: lessons must be learned from COVID-19. Lancet Glob Health.

[13] Martínez JI, Montalbán EG, Bueno SJ, Martínez FM, Juliá AN, Díaz JS, Marín NG, Deorador EC, Forte AN, García MA (2022). Monkeypox outbreak predominantly affecting men who have sex with men, Madrid, Spain, 26 April to 16 June 2022. Euro Surveill.

[14] Kmiec D, Kirchhoff F (2022). Monkeypox: a new threat?. Int J Mol Sci.

[15] Nuzzo JB, Borio LL, Gostin LO (2022). The WHO declaration of monkeypox as a global public health emergency. JAMA.

[16] Rao AK, Petersen BW, Whitehill F, Razeq JH, Isaacs SN, Merchlinsky MJ, Campos-Outcalt D, Morgan RL, Damon I, Sánchez PJ (2022). Use of JYNNEOS (smallpox and monkeypox vaccine, live, nonreplicating) for preexposure vaccination of persons at risk for occupational exposure to Orthopoxviruses: Recommendations of the Advisory Committee on Immunization Practices—United States, 2022. MMWR Morb Mortal Wkly Rep.

[17] Rizk JG, Lippi G, Henry BM, Forthal DN, Rizk Y. Prevention and treatment of monkeypox. Drugs. 2022; 1–7. 10.1007/s40265-022-01742-y PMC9244487 35763248

[18] Siegrist EA, Sassine J (2022). Antivirals with activity against monkeypox: a clinically oriented review. Clin Infect Dis.

[19] Huston J, Curtis S, Egelund EF. Brincidofovir: a novel agent for the treatment of smallpox. Ann Pharmacother. 2023; 10600280231151751. 10.1177/10600280231151751 36688308

[20] Desai AN, Thompson GR, Neumeister SM, Arutyunova AM, Trigg K, Cohen SH (2022). Compassionate use of tecovirimat for the treatment of monkeypox infection. JAMA.

[21] O’Laughlin K, Tobolowsky FA, Elmor R, Overton R, O’Connor SM, Damon IK, Petersen BW, Rao AK, Chatham-Stephens K, Yu P (2022). Clinical use of tecovirimat (Tpoxx) for treatment of monkeypox under an investigational new drug protocol—United States, May–August. MMWR Morb Mortal Wkly Rep.

